# Laser Alloying Advantages by Dry Coating Metallic Powder Mixtures with SiO_x_ Nanoparticles

**DOI:** 10.3390/nano8100862

**Published:** 2018-10-21

**Authors:** Michael C. H. Karg, Michael Rasch, Konstantin Schmidt, Sophia A. E. Spitzer, Till F. Karsten, Daniel Schlaug, Cosmin-Rudolf Biaciu, Andrey I. Gorunov, Michael Schmidt

**Affiliations:** 1 Institute of Photonic Technologies (LPT), Friedrich-Alexander-Universität Erlangen-Nürnberg (FAU), Konrad-Zuse-Straße 3/5, 91052 Erlangen, Germany; michael.rasch@lpt.uni-erlangen.de (M.R.); konstantin.schmidt14@gmx.net (K.S.); sophia.spitzer@gmx.de (S.A.E.S.); felixkarsten@icloud.com (T.F.K.); daniel.schlaug@web.de (D.S.); rudi.biaciu@studium.fau.de (C.-R.B.); michael.schmidt@lpt.uni-erlangen.de (M.S.); 2 Collaborative Research Center 814-Additive Manufacturing (CRC 814), Friedrich-Alexander-Universität Erlangen-Nürnberg (FAU), Am Weichselgarten 9, 91058 Erlangen-Tennenlohe, Germany; 3 Erlangen Graduate School in Advanced Optical Technologies (SAOT), Friedrich-Alexander-Universität Erlangen-Nürnberg (FAU), Paul Gordan Straße 6, 91052 Erlangen, Germany; 4 Kazan National Research Technical University named after A.N. Tupolev-KAI, Karl Marx Str. 10, 420111 Kazan, Russia; gorunow.andrej@yandex.ru

**Keywords:** dry coating fumed silica nanoparticles, powder mixture segregation, in situ alloy creation, Selective Laser Melting™, Laser Metal Powder Bed Fusion, Aluminium Copper Magnesium alloys 2xxx, powder layer microscopy, EDX, tensile test

## Abstract

Up to now, minimizing segregation of free-flowing, microscale metal powder mixtures driven by different mass density is an open challenge. In this work, effects of particle size variation on homogeneity of Al-Cu mixtures, with a density ratio of 3.3, are examined. Dry coating Al particles with 0.3 wt% fumed silica SiO_x_ nanoparticles significantly decreases interparticle attraction. This enlarges the range of free-flowing Al particle sizes to < 20 µm. Powder mixture homogeneity is examined optically in vibrated bulk powder and thinly spread layers. From various powder mixtures, solid samples are built layer by layer with the Additive Manufacturing (3D printing) technology Laser Beam Melting in metal powder bed (LBM). Chemical homogeneity of solids is evaluated via energy-dispersive X-ray spectroscopy, backscattered electron microscopy, metallographic analysis and tensile tests. Persistent homogeneity of Al-Cu powder mixtures and LBM solids is found only with particles < 20 µm dry coated with SiO_x_ nanoparticles. Observed segregation phenomena are explained with a decrease in particle mobility at increasing local concentration and the decreasing effectiveness of mass in smaller particles. The main effects are based on geometry, so they are expected to be transferrable to other nanoparticles, alloying components and powder bed technologies, e.g., binder jetting.

## 1. Introduction

**Figure 1:**
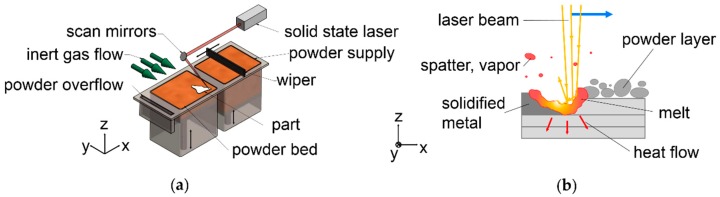
Laser Beam Melting in metal powder bed (LBM) fundamentals; (**a**) basic layout of an LBM machine; (**b**) basic interaction of laser beam (moving in direction of blue arrow), melt, powder and solidified metal in LBM.

Additive Manufacturing (AM) or synonymously 3D printing increasingly pervades industrial production [1]. An AM technology of growing scientific and industrial relevance is Laser Beam Melting in metal powder bed (LBM) [2,3]. LBM is one specific manifestation from the category of powder bed fusion processes [4,5]. The basic layout of an LBM machine is shown in Figure 1a. For each layer, powder is spread thinly by a wiper on top of the previous layer. The cross section of the product is melted with a laser beam, as shown in Figure 1b. Then, the platform is lowered by the layer thickness, Δ_z_. This cycle is repeated for each layer, until the product has been completely built. To ensure spreadability of powders in thin layers, typically spherical particles from atomization with inert gas are used. More details about the used LBM machine are explained in Section 2.

In many promising fields of application of AM, there is a need for more appropriate materials [3]. Characteristically in AM, materials, process conditions and system technology form strong bidirectional dependencies. Thus, to realize the full potential, proven materials from conventional manufacturing should not be transferred unquestioned, but be optimized with respect to the intended AM system and process [6]. This has sparked increasing research intensity in recent years, especially in materials science and technology. The response of novel alloys and metallic composites to the specific process conditions in LBM, including repeated melting and rapid solidification, are investigated. Frequent and controlled variation of chemical composition of powders is essential for experiments. An increasingly followed approach is the mixing of powders with different chemical composition. The components for mixtures, elementally pure powders and master alloys, are atomized in large scales. They are available in more reliable quality than custom atomized small batches of prealloyed powders, in terms of particle shape and impurity content [7,8], and of chemical composition of alloying elements [9,10,11]. However, powder mixtures tend to de-mix in motion if components differ in volumetric mass density. A commercially successful mixture of mainly Ni and CuSn10 particles had a density ratio of 1.035, but was not fully melted and truly alloyed due to system technology limitations at the time [12,13,14]. Mixtures of Fe and Cu with a density ratio of 1.133 were found to be less suitable due to segregation of components [15,16].

More recent publications on alloy creation in LBM from powder mixtures are summarized in Table 1, sorted by increasing density ratio, ρ_A_/ρ_B_, of the mixed components, A and B [17,18,19,20,21,22,23,24,25,26]. Often the focus of attention is set on microstructural details of novel alloy compositions exposed to the process conditions of LBM. Because effects of LBM process parameters depend on the powder composition, local de-mixing can prohibit reliable processing and cause decreased relative density, ρ_rel_ [20,21,22,25]. Especially towards larger density ratios, ρ_A_/ρ_B_, homogeneous distribution of alloying elements in LBM products is described as unsatisfactory [17,18,20,21,22,23,24,25]. Homogenizing effects of post-LBM heat treatments are mentioned [17,24]. Local inhomogeneity will pronouncedly show negative effects in tensile test results of Ultimate Tensile Strength (UTS) [20] and Elongation at break (E) [23], but not in compressive tests [25,27]. Examination of segregation phenomena in metal powders before LBM is deficient in published literature.

A generally applicable approach to avoiding segregation is avoiding the mixing of powders [28]. In AM, this is successfully practiced, e.g., in Laser Metal Deposition (LMD). LMD is a different AM process from the group of directed energy deposition [4]. In LMD, metal powder is fed into a melt pool with a gas stream. With separate streams feeding different powders into the same melt pool, no mixing of powder is required. The different materials mix in the melt. To transfer this approach to LBM or in general to technologies based on a powder bed, e.g., Electron Beam Melting or Binder Jetting [4], poses interesting challenges in re-design of system and process technology.

Focusing on the powder material, segregation can be reduced by limiting relative mobility of powder components. One method following this approach is mechanical alloying by high energy ball milling before LBM [29,30]. Results can be influenced with numerous parameters. Another method reducing relative mobility is coating nanoparticles on the surface of metal microparticles with different chemical composition. This can be achieved with ceramic nanoparticles in a sophisticated wet coating procedure: nanoparticles are suspended in water, deagglomerated with a short-pulsed laser beam, mixed with metal powder and furnace dried [31,32]. A dry coating procedure yielding similar results with metallic and ceramic nanoparticles on metallic microparticles has been demonstrated to be suitable for in situ alloying in LBM [33,34,35,36]. Instead, the focus of this work is set on mixtures of microscale particles with different chemical composition.

Another universal approach to avoiding segregation is decreasing overall mobility of particles by increasing powder cohesion, e.g., by adding liquids [37,38]. This is successfully applied to mixtures, e.g., in pharmacy, food processing or construction materials, but not applicable to LBM, because increasing cohesion decreases the ability of powders to be spread in smooth, thin layers. Additionally, liquids evaporate when metal is melted, and cause voids in the solidified product.

However, effects of interparticle attraction on segregation are not monotonous. Experiments with magnetic and non-magnetic particles of different density under varied strength of a magnetic field show segregation can be avoided, or the direction of segregation be inverted [39].

Particle Size Distribution (PSD) is another influential factor in de-mixing phenomena [40,41]. In experiments with smaller glass and larger steel balls on the millimeter scale, segregation can be avoided by compensating higher mass density with larger particle size [42].

No publications describe segregation of free-flowing, metal, microparticles suitable for LBM.

In a generalized way, mixing and de-mixing phenomena can be described independently of the experimental setup by the Fokker-Planck equation [37].

(1) $$m(x)=q(x)T(x)-\frac{\partial }{\partial x}{(q(x)D}^{*}(x))$$

It describes the change of concentration, q, of a mixture component at a location, x, by balancing incoming and outgoing particles. It can be transformed to express local mass flow of a mixture component, m, by a convective part dominated by the convective coefficient, T, and a dispersive part dominated by the dispersive coefficient, D* [37]. If convective transport is selective of mixture components, the stationary condition will be less than stochastically homogeneous [37]. Convection is typically realized by moving parts. In LBM, this can be the wiper that spreads powder layers. Additionally, powder can de-mix during LBM without convection due to vibration. A key requirement for stable mixtures in conditions without convection is the independence of particle mobility from q [37]. If increasing q correlates with reduced particle mobility, random accumulations of one component will grow and the mixture segregate.

Mobility of particles depends on particle sizes, e.g., smaller particles can move through gaps between larger particles. Gravity and inertia also depend on particle size. With metal particles in the single-digit and double-digit micrometer scale, effects of particle mass can be pushed into the background by surface effects [43]. One consequence of this is reduced powder flowability, which can be compensated sufficiently to facilitate spreading in thin layers for LBM by dry coating with nanoparticles [36,44,45,46]. Dry coated nanoparticles act as spacers increasing the distance between microparticles and lowering van der Waals forces [47].

The hypothesis put to experimental falsification in this work is: size variation of free-flowing metal microparticles does not allow stabilizing mixtures against segregation. The system of Al-Cu is chosen because of its challenging mass density ratio of 3.3, and because it attracts increasing scientific interest with regards to LBM [25,27,48,49,50,51,52,53,54,55,56,57,58,59,60], since processability has been shown [61,62]. The range of free-flowing particle sizes is extended to particles < 20 µm by dry coating with SiO_x_ nanoparticles [46]. Maximum particle size is limited to 63 µm, which is common for LBM. De-mixing is investigated with convective transport, during spreading of thin layers, and without convective transport, under vibration. Chemical homogeneity is investigated in solid LBM samples, and after subsequent heat treatment. Because they are sensitive to detect locally compromised mechanical properties resulting from inhomogeneities, tensile tests are conducted and analyzed. The alloy system is expanded to quaternary Al-Cu-Mg-Ti closer to the commercial alloy EN AW-2024, to increase complexity and for better comparability to a benchmark material.

## 2. Materials and Methods

**Figure 2:**
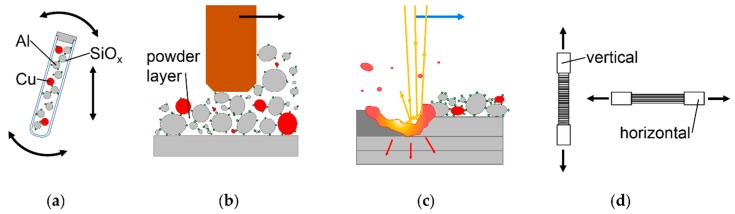
General methodology followed in this article; (**a**) evaluation of de-mixing in bulk powder with dry coated SiO_x_ and varied PSD; (**b**) evaluation of de-mixing in thin powder layers inside LBM machine; (**c**) LBM experiments with micrographic analysis of relative density, ρ_rel_, defects, hardness and chemical homogeneity; (**d**) tensile tests of vertically and horizontally built specimens.

The general methodology followed in this work is illustrated in Figure 2. Bulk powder mixing and segregation is examined by image analysis of vibrated test tubes, as shown in Figure 2a. This represents conditions during powder transport, e.g., inside an LBM machine. Cu distribution in thin powder mixture layers spread inside an LBM machine, as shown in Figure 2b, is analyzed via light microscopy, and contrasted to test tube vibration results. To compare the processability of different mixtures and achievable relative density, ρ_rel_, LBM experiments are conducted, as shown in Figure 2c. LBM samples are examined micrographically, via Back Scattered Electron (BSE) microscopy, Energy Dispersive X-ray spectroscopy (EDX), and hardness testing. To further evaluate the homogeneity of in situ alloyed LBM parts, tensile tests are conducted. To examine anisotropy, tensile specimens are built vertically, with layers orthogonal to the pulling direction, and horizontally, with layers parallel to the pulling direction, as shown in Figure 2d. To examine the homogenization of heat treatment, LBM products are analyzed as built and after subsequent T4 heat treatment.

Al powder atomized with N is sourced from Ecka Granules Germany GmbH (Velden, Germany), Cu atomized with N from TLS Technik GmbH & Co Spezialpulver KG (Bitterfeld, Germany), and a master alloy AlMg50 containing 50 wt% Mg atomized with N from Nanoval GmbH & Co. KG (Berlin, Germany). Pure Mg powder is avoided due to flammability. Prealloyed Ti6Al4V atomized with Ar is sourced from TLS Technik GmbH & Co Spezialpulver KG (Bitterfeld, Germany). At delivery, powders are imaged with a Philips XL Scanning Electron Microscope (SEM) (Koninklijke Philips N.V., Amsterdam, Netherlands), and chemical composition is analyzed by X-ray fluorescence spectroscopy (XRF) on a SpectroMAXx (SPECTRO Analytical Instruments GmbH, Kleve, Germany). All powders are sieved under Ar with 20, 32, 40, 50 and 63 µm wide meshes. For all experiments, except establishment of the initial situation and some angle of repose measurements, Al powders are dry coated with 0.3 wt% SiO_x_ fumed silica nanoparticles Aerosil^®^ R 106 by Evonik Industries AG (Essen, Germany). This is conducted by mixing in a Turbula^®^ T2A shaker (Willy A. Bachofen AG, Muttenz, Switzerland) on maximum speed for 1 h [46,63]. No ball milling and no mechanical alloying are performed. The angle of repose φ is determined in the style of ISO 4324 [64].

To establish the initial situation without SiO_x_ nanoparticles, as a reference point, and to demonstrate the challenges of in situ alloying in LBM, a powder mixture with eutectic composition 67.3 wt% Al and 32.7 wt% Cu is processed according to the current state of the art. Al and Cu are sieved to PSD 20–50 µm as established in LBM of Al [65,66].

Components of powder mixtures are weighed on a scale with 0.1 mg accuracy. They are mixed for minimum 2 h in a Turbula^®^ T2A (Willy A. Bachofen AG, Muttenz, Switzerland) under Ar, in a half-filled 15 cm high container. Powders for vibration experiments are mixed for 16 h in the T2A under Ar, in half filled 10 cm high test tubes. 25 binary mixtures of 94.7 wt% Al, 5 wt% Cu, and 0.3 wt% SiO_x_ are prepared by combining five particle size fractions < 20 µm, 20–32 µm, 32–40 µm, 40–50 µm and 50–63 µm. Based on the results, 81 quaternary powder mixtures are prepared. The chemical composition is chosen to approximate the high-strength and high-ductility alloy EN AW-2024 [67], which is identically standardized as AA 2024 [68], with 3.8–4.9 wt% Cu, 1.2–1.8 wt% Mg, 0.3–0.9 wt% Mn, and balanced Al. Mn is left out to limit experimental effort, because its effects are considered minor in LBM compared to the other elements [69]. The four powders are mixed to contain 94.0 wt% Al, 4 wt% Cu, 1.5 wt% Mg, 0.15 wt% Ti, and 0.3 wt% SiO_x_. The relatively high Ti content, still within the limits of EN AW-2024, is chosen to reduce crack tendency by grain refinement [55,69]. If the Si contained in 0.3 wt% SiO_x_ nanoparticles dissolves in the melt, it will not exceed the limit of 0.5 wt% for Si in EN AW-2024. Remaining O might form oxides with Ti, Al or Mg, which are more affine to O than Si [70]. 81 quaternary mixtures combine three particle size fractions < 20 µm, 20–32 µm, and 32–40 µm of four powders. 80 are evaluated, as one tube is broken. Bulk powder densities are determined in the style of ISO 3923 [71].

To examine segregation in bulk powder, test tubes are vibrated for 2.3 h with an amplitude of 0.5 mm at a frequency of 50 Hz. Tubes are mounted with a jig on a vibration sieve EML 200 digital plus (HAVER & BOECKER OHG, Oelde, Germany). Segregation levels are categorized visually by the strong optical contrast between reddish Cu and grey Al particles. Because contrast between bright grey Al, dark grey Ti6Al4V, and dark grey AlMg50 is lower, duration of vibration is extended to 5.25 h for quaternary mixtures. Segregation of darker powders cannot be distinguished between Ti6Al4V and AlMg50. However, striations and separated dark bands at top and bottom of tubes are clearly visible against bright grey Al.

**Figure 3:**
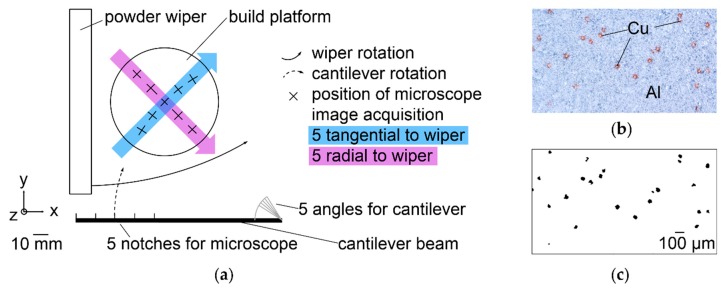
Determination of mixture homogeneity in thin powder layers with a microscope inside the LBM machine; (**a**) setup and imaging positions along and across the direction of powder spreading; (**b**) image with reddish Cu particles contrasting to grey Al (**c**) the same image in black and white.

Powder layer spreading and LBM experiments are conducted on a commercially available LBM machine, SLM^®^ 50, by manufacturer ReaLizer GmbH (Borken, Germany). SLM 50 has a rotational wiper with two silicone rubber lips, working in turns to move powder back and forth over the build platform. It does not drop a portion of powder into an overflow vessel each layer. A constant rotational wiper velocity is set. It yields a minimum tangential velocity of 12.4 mm/s at the edge of the platform closest to the rotational axis of the wiper and a maximum of 21.8 mm/s. Potential effects of different wiper velocities can be detected along the radial direction marked with a violet arrow in Figure 3a. Images are taken after the wiper has passed the platform in the direction marked with a blue arrow in Figure 3a. A CMOS microscope dnt DigiMicro Profi (Drahtlose Nachrichtentechnik Entwicklungs- und Vertriebs GmbH, Dietzenbach, Germany) with a resolution of 2592 × 1944 pixels is mounted on a cantilever beam, which can be swung over the build platform. Nine positions for imaging are reproducibly reached as illustrated in Figure 3a. This allows analysis of powder layer homogeneity radially and tangentially to the rotation of the wiper. Along the vertical Z axis, every fifth of 100 layers is imaged at Δ_z_ = 30 µm. Because of strong deviations in the first two imaged layers above the build platform, only the following 18 are analyzed. The first layers of LBM typically form support structures that are not part of the product. The Al build platforms are roughened by blasting with Al_2_O_3_ particles before use. Figure 3b shows a detail of an obtained image, Figure 3c the same after processing and conversion to black and white.

Three binary mixtures with 94.7 wt% Al, 5 wt% Cu, and 0.3 wt% SiO_x_ are analyzed with the setup in Figure 3. They have different PSD: firstly, Al < 20 µm and Cu < 20 µm, secondly, Al < 20 µm and Cu 40–50 µm, and thirdly, Al 20–32 µm and Cu < 20 µm. The different PSD lead to varied visibility of Cu particles in microscope images. Larger Cu particles have a higher probability of sticking out the thin powder layers than smaller ones, which may be hidden below Al particles. Additionally, deviations of pixel size originating from particle edges depend on PSD. Therefore, the absolute values of black pixels representing Cu can only be compared within a given PSD. The three PSD are compared by ratios of local to global means. Local means are calculated differently for the three spatial directions.

(2) $$\bar{{\mathrm{Cu}}_{t,r}}=\frac{\sum_{i=1}^{18}{\mathrm{Cu}}_{i}}{18}$$

For tangential and radial directions, nine different local means, $\bar{{\mathrm{Cu}}_{t,r}}$, are calculated, each from 18 images acquired at the same X, Y-position on the platform over 18 layers. In the vertical, 18 local means, $\bar{{\mathrm{Cu}}_{v}}$, are calculated, each from nine images of a layer. Global mean, $\bar{{\mathrm{Cu}}_{g},}$ is calculated, as the mean of 162 images acquired at nine different locations on the platform in 18 layers.

(3) $$\bar{{\mathrm{Cu}}_{v}}=\frac{\sum_{j=1}^{9}{\mathrm{Cu}}_{j}}{9}$$

(4) $$\bar{{\mathrm{Cu}}_{g}}=\frac{\sum_{i=1}^{18}\sum_{j=1}^{9}{\mathrm{Cu}}_{i,j}}{18\times 9}$$

The LBM machine Realizer SLM^®^ 50 (ReaLizer GmbH, Borken, Germany) has a single mode Yb fiber laser YLM-100-AC from IPG Photonics (Oxford, MA, USA) specified with 1070 nm wave length, linewidth of < 2.5 nm, maximum output power, *P*, of 100 W, random polarization, and a beam quality factor, *M*², of 1.05–1.1. It is operated in continuous wave mode. The laser beam is guided over the X, Y-plane with two galvanometer scanners from Cambridge Technology (Bedford, MA, USA). Scan speed is set in the machine control software by point distance, *d*_p_, and exposure time, *t*_e_. Actual *v*, which is determined as mean of 6 calibrated high-speed camera measurements made with a Phantom V1210 (Vision Research, Wayne, NJ, USA) for each used combination of *d*_p_ and *t*_e_, is found to deviate up to 46% from theoretical scan speed *v*_theo_, that is calculated, and the relative deviations are found to depend only on *t*_e_.

(5) $$v_{\mathrm{theo}}=\frac{d_{p}}{t_{e}}$$

**Figure 4:**
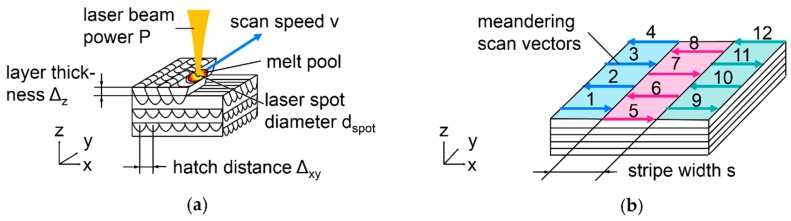
LBM parameters and scan strategies; (**a**) alternating meander scanning with opposite direction of neighboring scan vectors and 90° rotation around Z-axis each layer; (**b**) striped scanning.

In this work, only values for actual v, as confirmed by measurements, are mentioned. The laser beam is focused with an F-θ lens to ensure comparable intensity at different positions on the build platform. Constantly set spot diameter in the processing plane, *d*_spot_, of 66 µm, *P* = 100 W, and rotationally symmetrical Gaussian intensity distribution are confirmed by measurements with a MicroSpotMonitor (PRIMES GmbH, Pfungstadt, Germany). For LBM experiments, Ar is used as process gas to remove vapor and spatter from the interaction zone of the laser beam with the metal. Before the laser can be activated, the process chamber is flushed with Ar and the O content decreased below 1%. The build platform heating is set to the maximum 200 °C. Thermocouple measurements on the surface reveal local variations of the actual temperature from 112 to 124 °C across the platform, when a plateau has been reached ten minutes after heating has begun. The varied LBM parameters are illustrated in Figure 4a: scan speed, v, and hatch distance, Δ_xy_. Laser power, *P* = 100 W, spot diameter *d*_spot_ = 66 µm, and layer thickness, Δ_z_ = 30 µm, are kept constant.

LBM parameters for establishment of initial situation in Section 3.2 are *v* = 650 mm/s and Δ_xy_ = 25 µm. Alternating meanders are scanned, with double exposure of each layer.

(6) $$E_{\mathrm{vol}}=\frac{P}{{v\times \Delta }_{\mathrm{xy}}{\times \Delta }_{z}}$$

From these parameters, volumetric energy density, *E*_vol_, is calculated, equivalent to 410 J/mm³.

LBM sample cubes have 5 mm edge length and 2 mm high supports designed in Magics V19.0.2.08 (Materialise NV, Leuven, Belgium). All cubes are built with alternating meander scan strategy as illustrated in Figure 4a: neighboring scan vectors have opposite direction. Each layer, scan directions are rotated by 90° around the vertical Z-axis. For tensile testing, cylinders with 7 mm diameter and 55 mm length are built vertically and horizontally on the platform, to investigate anisotropy. To ensure consistent ρ_rel_ in horizontal cylinders, maximum scan vector length is limited [56,72,73] by striped scanning with stripe width, s, of 1 mm. As schematically illustrated in Figure 4b with scan vectors numbered in the order of their exposure, each stripe is filled with meandering vectors before the next stripe is scanned. Each layer, stripes and vectors are rotated by 90° around Z. Half the tensile samples are T4 heat-treated, i.e., solution annealed for 3 h at 500 °C in a chamber furnace N11/HR (Nabertherm GmbH, Lilienthal, Germany) quenched in ice water, and aged at room temperature for minimum 5 days. All tensile samples are lathed into testing shape and threaded M6 following DIN 50125 B 4 × 20 [74]. Tensile testing at room temperature is performed according to ISO 6892-1 [75].

For micrographic preparation, LBM samples are embedded in resin, ground, and polished with subsequently finer grain size down to 1 µm. Samples made from the quaternary powder mixture are additionally lapped, to lay bare fine cracks by removing grinding debris with free-rolling abrasive 9 µm diamond particles. After lapping, polishing is continued down to 1 µm grain size. Analysis of stitched high-resolution optical microscope Aristomet (Leica Microsystems GmbH, Wetzlar, Germany) images covering full sections of LBM samples, with exception of the rough outer edges, delivers ρ_rel_ by thresholding and conversion to black and white following VDI 3405 Blatt 2 [76]. Vickers Hardness measurements are performed on a KB 30 S (KB Prüftechnik, Hochdorf-Assenheim, Germany) according to ISO 6507-1 [77]. For analysis of chemical homogeneity via BSE and EDX, a Zeiss Merlin SEM (Carl Zeiss Microscopy GmbH, Jena, Germany) with Oxford Instruments EDX detector (Abingdon, UK) is used. As a benchmark for alloy homogeneity, a sample of EN AW-2024 from conventional manufacture by extrusion is compared. It has been T4 heat-treated identically to the LBM samples. Three EDX line measurements of 2 mm length are evaluated per sample. Each line consists of 500 data points. EDX lines are oriented in parallel to buildup direction Z and the direction of extrusion of the conventional sample. Quaternary LBM samples for analysis of grains in Section 3.8 are etched with 100 mL H_2_O, 0.8 mL HNO_3_, 0.25 mL HCl, and 0.75 mL HF. The sample from the initial situation in Section 3.2 is etched with 100 mL H_2_O, 2.6 mL HNO_3_, 0.9 mL HCl, and 0.2 mL HF. Grain sizes of in situ alloyed Al-Cu-Mg-Ti are determined from etched T4 samples in the style of ISO 643 [78]. Micrographs of two vertical and two horizontal samples each cut in parallel to buildup direction Z and orthogonal to it are used to determine mean grain size and aspect ratio.

## 3. Results and Discussion

### 3.1. Incoming Inspection of Metal Powders

**Figure 5:**
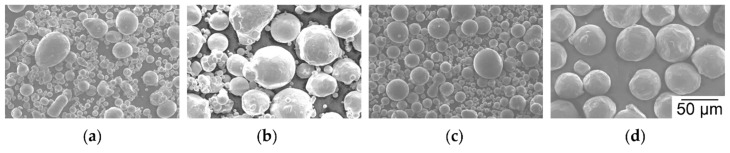
SEM images of spherical powder particles as delivered before sieving; (**a**) N atomized Al; (**b**) N atomized Cu; (**c**) N atomized AlMg50; (**d**) Ar atomized Ti6Al4V.

SEM images show the powders as procured before sieving. They have mostly spherical to slightly ovoidal particle shapes. That indicates atomization with inert gas and little O absorption, which would cause spattered particles [46,79]. Increased O content impedes high ρ_rel_ in LBM of Al alloys [8,36]. Mostly spherical shape increases accuracy of fractioning particle sizes by sieving compared to elongated particle shape. Elongated particles can pass through sieve meshes that are narrower than the larger axis of the particles. PSD appear similar for Al in Figure 5a, Cu in Figure 5b, and AlMg50 in Figure 5c. Ti6Al4V in Figure 5d shows fewer fine particles and a narrower PSD. Reasons can be the different systems, process settings, and gases used for atomization, as well as fractioning according to size by the powder suppliers. However, the very small number of particles in each SEM image compared to the number of particles required for experiments limits the value of statements. Further details about the PSD after sieving are explained in the Appendix A.

The chemical analysis of powders listed in Table 2 shows only 99.7 wt% purity of Al. As this is the main mixture ingredient, it will impact results. Ti6Al4V also deviates from expectation, but will affect the mixtures much less because of the low amounts that are used of this powder.

### 3.2. Experimental Establishment of the Initial Situation in LBM of Al-Cu Powder Mixtures Without SiO_x_

**Figure 6:**
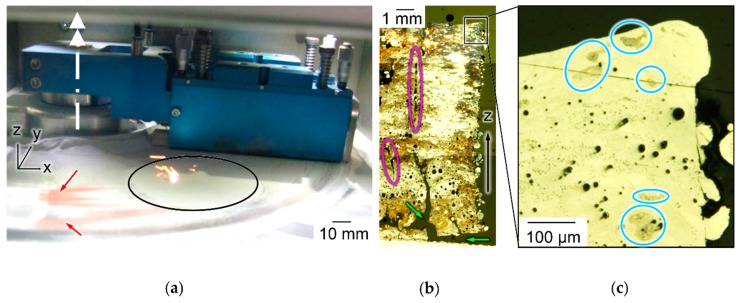
Challenges of in situ alloying Al-Cu powder mixtures in LBM, experimental establishment of initial situation with default parameters and PSD without SiO_x_; (**a**) Cu segregation from thin powder layer inside LBM machine (red arrows), build platform (black), wiper axis (white); (**b**) etched section of LBM sample with irregular color, thin vertical defects (pink), delamination from platform (green arrows); (**c**) detail with incompletely melted Cu particles (blue) and spherical pores (dark).

To exemplify challenges of in situ alloying Al-Cu powder mixtures in LBM without SiO_x_ nanoparticles, samples are built with powder and process parameters suitable for prealloyed Al-Cu. Results are illustrated in Figure 6. During spreading of thin powder layers, segregation of reddish Cu particles is clearly visible in contrast to grey Al in Figure 6a. Cu is increasingly deposited at the turning point of the blue wiper outside the build platform. The etched micrograph of a sample in Figure 6b shows irregular color over build height Z and within layers, indicating uncontrollable variation of chemical composition. Various types of defects appear: vertically elongated areas with incomplete melt coalescence highlighted in pink ellipses, delamination from the build platform highlighted with green arrows and dark spherical pores. Higher magnification of the same sample before etching shows spherical pores in Figure 6c. They could have been caused by high melt pool dynamics resulting from excess energy input or by entrapped hydrogen, released from Al at solidification [80]. Comparing Figure 6b,c, pore size and local distribution appear irregular. The blue ellipses in Figure 6c highlight incompletely melted Cu particles of irregular shape and random location. The irregular occurrence of diverse types of defects under constant LBM parameters indicates uncontrolled variation of powder composition along all spatial directions.

### 3.3. Angle of Repose φ of Different Powders and Particle Size Fractions with and without Dry Coated SiO_x_

**Figure 7:**
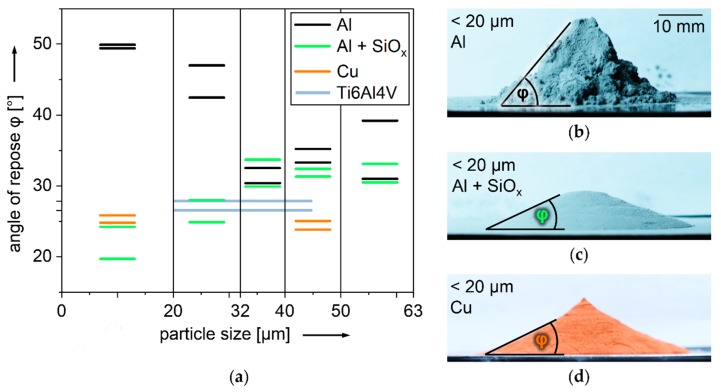
Lowering cohesion by dry coating SiO_x_ nanoparticles, visualized with angle of repose in the style of ISO 4324 [64]; (**a**) comparison of powder fractions; (**b**) pile of Al < 20 µm, φ = 50°; (**c**) pile of Al < 20 µm dry coated with 0.3 wt% SiO_x_ nanoparticles, φ = 24°; (**d**) pile of Cu < 20 µm, φ = 26°.

A visually tangible comparison of the flowability of different powder particle size fractions according to the angle of repose φ, in the style of ISO 4324, is shown in Figure 7a. As a reference for powder with proven suitability for LBM [81,82,83], Ti6Al4V 20–45 µm is shown in light blue. Insufficient flowability correlates with inhomogeneous spreading in thin powder layers and volume defects in LBM products [46,84,85]. All Al powders, represented in black lines, have higher φ than TiAl64V, indicating lower flowability. The most cohesive is the Al powder fraction < 20 µm, reaching φ = 50°. The corresponding pile of powder is shown in Figure 7b. It has steep, cliffy slopes of irregular shape. Al particles < 20 µm are known to impede flowability and spreading in smooth thin layers. They decrease ρ_rel_ of LBM samples [46,81,85]. The lowest φ of uncoated Al is reached by the fraction 32–40 µm, but it is still slightly higher than Ti6Al4V. This is the preferred PSD for LBM of Al [65,66]. Al powder reaches significantly lower φ if dry coated with 0.3 wt% SiO_x_, as shown in light green in Figure 7a [46]. The largest drop in φ of 25–30° caused by dry coating SiO_x_ is observed with Al < 20 µm, falling below Ti6Al4V particles. The corresponding pile is shown in Figure 7c. Cu can be considered similarly free-flowing in small and large particle sizes. It reaches lower φ than Ti6Al4V 20–45 µm in the PSD < 20 µm, the corresponding pile is shown in Figure 7d, as well as in the PSD 40–50 µm. It has been shown that dry coating with SiO_x_ nanoparticles can significantly improve flowability of Al particles < 20 µm, and thus enable the spreading of smooth layers and LBM with high ρ_rel_ [46]. This expands PSD range for LBM of Al.

### 3.4. Segregation Behavior of Free-flowing Powder Mixtures with SiO_x_ and Varied PSD in Vibrated Test Tubes

**Figure 8:**
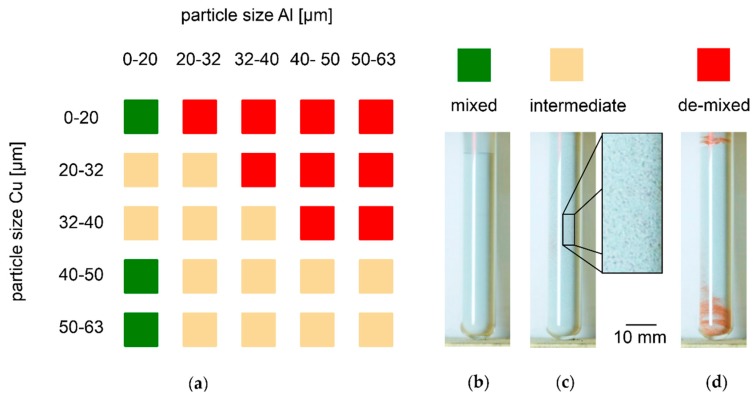
De-mixing of Al-Cu powder mixtures under variation of particle size fractions from test tube experiments, 94.7 wt% Al, 5 wt% Cu, and 0.3 wt% SiO_x_; (**a**) results; (**b**) example of powder remaining mixed; (**c**) example of intermediate powder with local Cu accumulations; (**d**) example of de-mixed powder with clearly visible segregation of Cu particles on top and bottom of the test tube.

The results of test tube experiments with binary Al-Cu powder mixtures are summarized in Figure 8a. Exemplary results of the three qualitatively distinguished grades of de-mixing are shown in Figure 8b–d. Samples with no visible de-mixing are categorized as mixed and represented in green squares. Figure 8b shows an example of homogeneously grey powder. Samples with highly visible de-mixing of reddish Cu powder on the test tube bottom, top, or both, are categorized as de-mixed. Figure 8d shows an example of a test tube with segregated Cu on top and bottom. De-mixed samples are represented by red squares in Figure 8a. Samples that reveal local accumulations of Cu particles throughout the height of the test tube, shown in Figure 8c at larger magnification, are categorized as intermediate, and represented in beige squares. All mixtures with particle sizes Al > Cu de-mix. Only samples including Al particles < 20 µm remain mixed. These are the mixtures with both Al and Cu particles < 20 µm and those with Cu particles > 40 µm. The remaining fall into the intermediate category. During the experiments, it is observed that most mixtures categorized as de-mixed after vibration already show some degree of segregation after the mixing procedure. An exception is mixtures containing Cu particles 32–40 µm: they only de-mix during vibration. The mixtures ending up in the intermediate category appear fully mixed before vibration. These results confirm that creation of reliably stable Al-Cu powder mixtures is probably not possible with particle sizes > 20 µm that are established in LBM of Al without SiO_x_.

**Figure 9:**
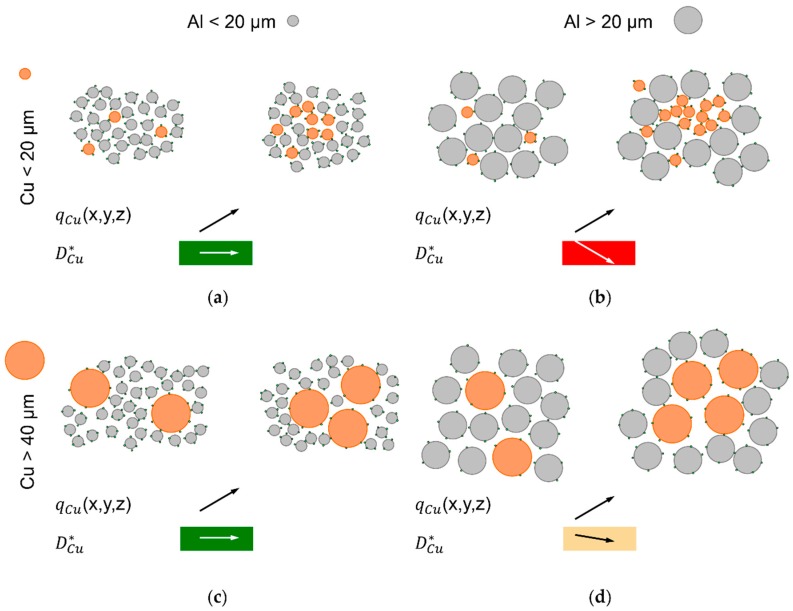
Considerations of changing mobility of Cu particles, $D_{\mathrm{Cu}}^{*}$, depending on local Cu concentration, *q*_Cu_, and different particle sizes, in vibrated bulk; (**a**) Al and Cu < 20 µm; (**b**) Al > 20µm and Cu < 20 µm; (**c**) Al < 20 µm and Cu > 40 µm; (**d**) Al > 20 µm and Cu > 40 µm.

To explain these observations, the dispersive coefficient of Cu, $D_{\mathrm{Cu}}^{*}$, is qualitatively compared between lower and higher Cu concentrations, *q*_Cu_. $D_{\mathrm{Cu}}^{*}$ is a measure for mobility of Cu particles among the majority of Al. Motion of particles is provided by vibration of the bulk powder. Decreasing $D_{\mathrm{Cu}}^{*}$ with increasing *q*_Cu_ causes segregation [37]. Four cases are distinguished, as illustrated in Figure 9a–d.

With all particles < 20 µm, the experimental result of no segregation leads to the assumption that $D_{\mathrm{Cu}}^{*}$ remains constant with increasing *q*_Cu_, as shown in Figure 9a. Al and Cu particles have similar size, but different mass. To explain the experimental result, it seems most plausible to assume that different mass has no significant effect in this case, which is characterized by small particle sizes < 20 µm and high flowability ensured by dry coating with SiO_x_ nanoparticles.

The second case of Al > 20 µm and Cu < 20 µm is illustrated in Figure 9b. The smaller Cu particles fit in gaps between the larger Al particles, which grants them higher $D_{\mathrm{Cu}}^{*}$ than among other Cu particles of similar size. Higher *q*_Cu_ increases chances that near gaps between larger Al particles are occupied by other Cu particles, which further decreases $D_{\mathrm{Cu}}^{*}$. Assuming mass has less effects at this size of Cu particles is supported by the observation that Cu accumulations on top of test tubes are more pronounced than with larger Cu particles. Gravity and smaller particle size could be expected to result in downward transport of Cu under vibration [38,40,42].

In the case of Al < 20 µm and Cu > 40 µm, which is illustrated in Figure 9c, the experimental results show no segregation, which implies no reduced $D_{\mathrm{Cu}}^{*}$ at increased *q*_Cu_. This seems plausible, considering that the smaller Al particles, which are much more numerous than Cu particles in the investigated mixtures with 95 wt% Al, should be able to move into gaps between larger Cu particles. This way, they can separate Cu particles from each other. Since mass is expected to have significant effects at this size of Cu particles, it is apparently compensated by the smaller size of Al particles. A similar observation of stability under motion without convective transport is described for a mixture of 2.5 mm large glass balls with 3.5 mm steel balls, at a mass density ratio of 2.6 [42].

The fourth considered case is that of intermediate de-mixing observed for Al particles > 20 µm and Cu particles of the same or larger size, as illustrated in Figure 9d. Theory implies that $D_{\mathrm{Cu}}^{*}$ is slightly lower at higher *q*_Cu_, but not sufficiently lower to cause strong de-mixing. Due to the smaller difference in particle size compared to Figure 9c, the separating effect is expected to be weaker. Since mass should be significant in these Cu particle sizes, it may be speculated that a higher probability for collisions between Cu particles would reduce their average mobility compared to lower *q*_Cu_, which increases probability for Cu particles to collide with lighter Al particles of lower momentum.

**Figure 10:**
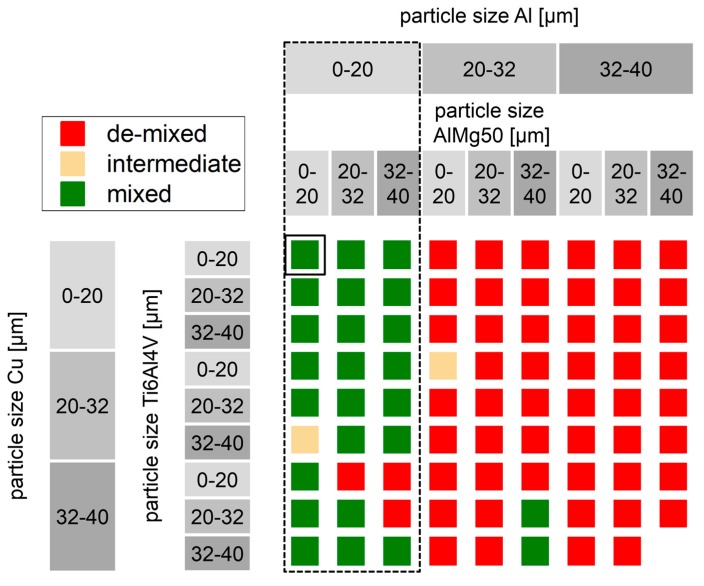
De-mixing of Al-Cu-Mg-Ti powder mixtures, under variation of particle size, obtained from vibrated test tubes, constant powder composition 92.4 wt% Al, 4 wt% Cu, 3 wt% AlMg50, 0.14 wt% Ti6Al4V, and 0.3 wt% SiO_x_; dominating factor for stability is Al particle size < 20 µm.

Results of test tube experiments with quaternary Al-Cu-Mg-Ti powder mixture are put together in Figure 10. The same color coding is used as with binary mixtures in Figure 8. In direct comparison, de-mixing is less pronounced in quaternary mixtures than in binary mixtures. Al particle size < 20 µm is the dominating factor for stable mixtures indicated by green squares. 23 out of 27 mixtures containing Al < 20 µm remain mixed. Only two of 53 mixtures without Al < 20 µm remain mixed. The reasons for the deviating results might be in the inaccuracies of the evaluation procedure. Powders are fractioned by sieving, which results in overlapping PSD, as further detailed in the Appendix A. After mixing and prior to vibration, no segregation of Ti6Al4V and AlMg50 can be found. After vibration, many mixtures show dark grey striations of Ti6Al4V or AlMg50 powder. Also, dark grey powder segregated on top or at the bottom of test tubes can be distinguished from light grey Al. Visual contrast is less than with reddish Cu powder, but clear enough for unambiguous categorization. Contrast could be improved by artificial illumination with light of wavelength ranges adapted to the specific reflectivity of the involved powders.

Mixtures with all particles sieved < 20 µm are considered most promising for controllably homogeneous in situ alloying.

### 3.5. Light Microscopic Analysis of Mixture Homogeneity in Thin Powder Layers

**Figure 11:**
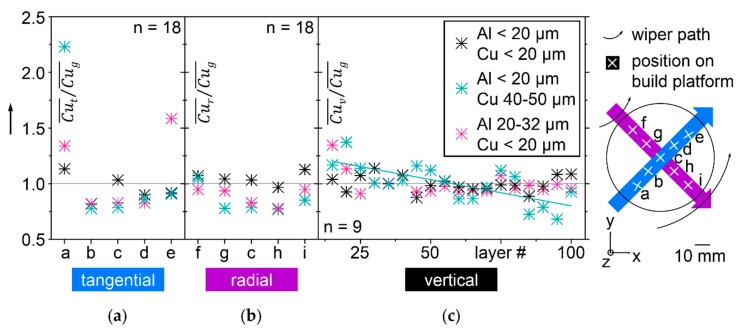
Homogeneity of Cu distribution compared by the ratios of local to global mean values for three binary Al-Cu powder mixtures with different PSD, determined by light microscopy of 60 µm powder layers in an LBM machine in three spatial directions; (**a**) tangential; (**b**) radial; (**c**) vertical.

The results of thin powder layer microscopy inside the LBM machine Realizer SLM 50 are refined in Figure 11. As a criterion to compare the homogeneity of binary Al-Cu mixtures with three different PSD, ratios of local and global means are calculated from the acquired light microscopic images, as described in Section 2 in detail and visualized in Figure 3. A hypothetical perfectly homogeneous mixture would only show ratios equal to one.

In Figure 11a, labelled tangential, homogeneity of the three different PSD along the spreading direction of the rotating wiper is compared. The mixture with Al and Cu particles < 20 µm shows values closest to one, indicating best homogeneity. This agrees with the results of vibration experiments. The mixture with larger Cu particles 40–50 µm that showed no segregation under vibration leads to pronounced accumulation of Cu particles at location a, which is hit first by the wiper. The other locations, b–e, consequently suffer from reduced Cu content. The third mixture with larger Al particles 20–32 µm and Cu particles < 20 µm shows increased Cu in positions a,e. Position e is the last in direction of the wiper. This mixture segregated in vibrated test tubes.

In Figure 11b, labelled radial, homogeneity along the length of the wiper is compared. Since the wiper in SLM 50 does not move translational but rotational, the tangential velocity of the wiper rises linearly from 12.4 mm/s at the edge of the platform closest to the rotational axis of the wiper to 21.8 mm/s at the opposite edge. In position f, the microscope imaged powder that was more slowly spread than in the positions following along the purple arrow in the legend of Figure 11. At least within this range of wiper velocities, only small effects on Cu distribution can be found. Again, the most homogeneous distribution of Cu is found in the mixture with all particles < 20 µm.

The comparison of Cu distribution between different layers spread subsequently at different vertical Z height over the build platform is illustrated in Figure 11c over the label vertical. It confirms the mixture with all particles < 20 µm as the most homogeneous one. The largest deviations from a ratio of means of one are found with Al particles < 20 µm and Cu 40–50 µm, a mixture that has been stable against segregation in vibrated bulk. With increasing Z height, optically detectable Cu in layers of this mixture decreases. A fitted teal line in Figure 11c indicates this tendency. The mixture with larger Al than Cu particles shows less variation of Cu content over the number of layers than the one with larger Cu particles, but more than the mixture with all particles < 20 µm.

In summary, the best homogeneity in vibration as well as spreading of thin layers is found in the mixture with all particles < 20 µm dry coated with SiO_x_. Segregation results in thin layers differ significantly from the results of vibrated test tubes. The mixture with larger Cu particles, which is stable under vibration, shows more segregation in thin layers than the mixture with larger Al particles, which is less stable under vibration. Conditions for de-mixing during spreading of thin layers differ significantly from vibrated bulk powder. They can be characterized by the moving wiper, that appears to selectively transport Cu by convection [37]. This selective transport is pronounced for the mixture with larger Al and the mixture with larger Cu. The mixture with all particles < 20 µm is least affected by this selectivity and stays most homogeneous in comparison. Uniform particle size in a range that decreases effect of particle mass appears to be beneficial for stability against de-mixing during spreading of thin layers. The mixture with larger Cu showed highest Cu content at the location closest to the turning point of the wiper, which resembles the results with all particles > 20 µm without SiO_x_, as shown in Figure 6a. The mixture with larger Al shows highest Cu content at location e, which is the furthest from the turning point of the wiper. A second, less pronounced peak of Cu content of the mixture with larger Al is found at location e, closest to the turning point of the wiper. Based on the current results, it cannot be excluded that another combination of particle size and mass may compensate for the selectivity of convective transport. This would probably be a powder mixture that is not stable against de-mixing under vibration, as the mixture with Al < 20 µm and Cu 40–50 µm segregated. To better understand the details of de-mixing phenomena during spreading of thin layers, it might be revealing to capture the powder movements in front of the wiper, in videos resolving single particles. Layer thickness, wiper geometry, and wiper velocity appear as probable influencing factors.

### 3.6. Micrographic Definition of LBM Process Maps

**Figure 12:**
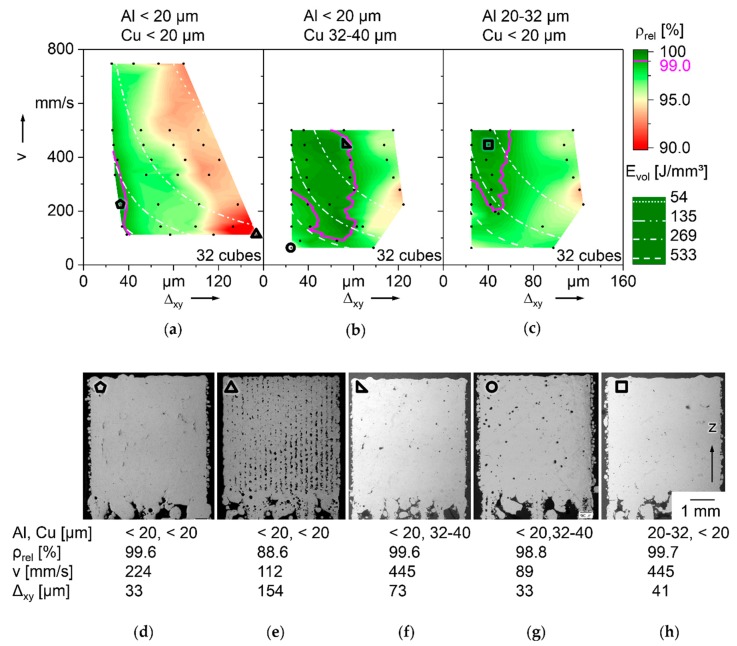
LBM process maps of Al-Cu powder mixtures with three different PSD, constant composition 94.7 wt% Al, 5 wt% Cu, and 0.3 wt% SiO_x_; (**a**–**c**) ρ_rel_ over v and Δ_xy_, pink contour ρ_rel_ = 99%, E_vol_ isolines in white; (**a**) Al and Cu particles < 20 µm; (**b**) Al < 20 µm, Cu 32–40 µm; (**c**) Al 20–32 µm, Cu < 20 µm; (**d**–**h**) exemplary micrographs; (**d**) few defects; (**e**) Δ_xy_ too large; (**f**) few defects; (**g**) many spherical gas pores at too high *E*_vol_; (**h**) few defects.

Experimentally defined process windows for binary Al-Cu powder mixtures with constant composition of 94.7 wt% Al, 5 wt% Cu, and 0.3 wt% SiO_x_ in three different PSD are visualized in Figure 12. Figure 12a–c show ρ_rel_ in colors shaded from red, for 90%, to dark green, for 100%. The dependence of Δ_xy_ is depicted along the horizontal and of v along the vertical axis. Other process parameters, P = 100 W, Δ_z_ = 30 µm, and d_spot_ = 66 µm, are kept constant. The white lines in Figure 12a–c designate constant E_vol_, as defined in equation (6). Pink contours mark ρ_rel_ = 99%. For each PSD, 32 cube samples of 5 mm edge length have been built on filigree supports and analyzed micrographically for ρ_rel_ and details of defects. In Figure 12a–c, each cube is represented by a black dot. The colors in between dots are interpolated. Selected micrographs are shown in Figure 12d–h, each marked with a small black symbol (pentagon, triangle, circle or square), which tags the respective data point in Figure 12a–c. Three combinations of particle size fractions of Al and Cu are chosen, one with all particles < 20 µm, illustrated in Figure 12a,d,e, one with larger Cu, illustrated in Figure 12b,f,g, and one with larger Al particles, as shown in Figure 12c,h. With all three mixtures, ρ_rel_ > 99.5% have been achieved. Examples of such cubes are shown in Figure 12d,f,h. From the other micrographs, reasons for lower ρ_rel_ can be concluded. Figure 12e with ρ_rel_ = 88.6% shows voids elongated in parallel to Z of approximately constant width and distance. The used Δ_xy_ = 154 µm must be too large for reliable coalescence. *E*_vol_ = 193 J/mm³ alone cannot give a satisfactory explanation, as lower 179 J/mm³ suffices for higher ρ_rel_ = 96.6%, at Δ_xy_ = 56 µm and *v* = 334 mm/s. An example for too high *E*_vol_ = 1135 J/mm³ resulting in less than optimal ρ_rel_ = 98.8% is shown in Figure 12g. The spherical pores typically result from excessive energy input causing increased melt pool turbulence. As a potential alternative, they might result from entrapped hydrogen [80].

The process window yielding ρ_rel_ > 99 % of the mixture of Al and Cu particles <20 µm that proved most stable against de-mixing is smaller than of the other two mixtures and shifted towards higher *E*_vol_. The powder mixture with smaller Cu particles than Al has a smaller range of LBM parameters yielding ρ_rel_ > 99% than the one with larger Cu particles, shifted towards smaller Δ_xy_. A potential approach for explaining this can be differences in density of powder layers caused by the different PSD [86,87], which is known to affect ρ_rel_ [88,89]. The bulk densities of the three mixtures determined according to ISO 3923 [71], as means of three measurements, are:

(1) Al and Cu < 20 µm: 1.42 g/cm³
(2) Al < 20 µm and Cu 32–40 µm: 1.45 g/cm³
(3) Al 20–32 µm and Cu < 20 µm: 1.47 g/cm³

The mixture with the smallest bulk density does show the smallest process window for high ρ_rel_. However, the one with the largest bulk density has a smaller process window than the one with intermediate bulk density. However, bulk powder density is not identical and not directly transferrable to density of thin powder layers [84,90]. The process windows for high ρ_rel_ might extend further with smaller Δ_xy_, which is prohibited by the LBM machine control software.

Another factor contributing to the rather small process window of the mixture with all particles < 20 µm may be a storage duration extended by several months before LBM compared to the two other mixtures. It may have increased oxidation. Surface oxide layers are known to impede melt coalescence in welding of Al [91]. They should have stronger effects in LBM, leveraged by larger surface area of particles. Figure 12d shows fine defects that may be the result of incomplete fusion, promoted by oxidation. Such defects are found in most micrographs of the mixture with all particles < 20 µm, but rarely in the two others processed within a few weeks after powder delivery.

No significant differences are found in hardness of samples built from the three mixtures, tested at n = 27 indentations per mixture, and more than seven days after LBM, to ensure natural ageing as a potential outcome of the largely undefined thermal history of LBM has reached a plateau:

(1) Al and Cu < 20 µm: 65.9 HV0.1 ± 6.2
(2) Al < 20 µm and Cu 32–40 µm: 68.4 HV0.1 ± 7.5
(3) Al 20–32 µm and Cu < 20 µm: 66.5 HV0.1 ± 5.6

**Figure 13:**
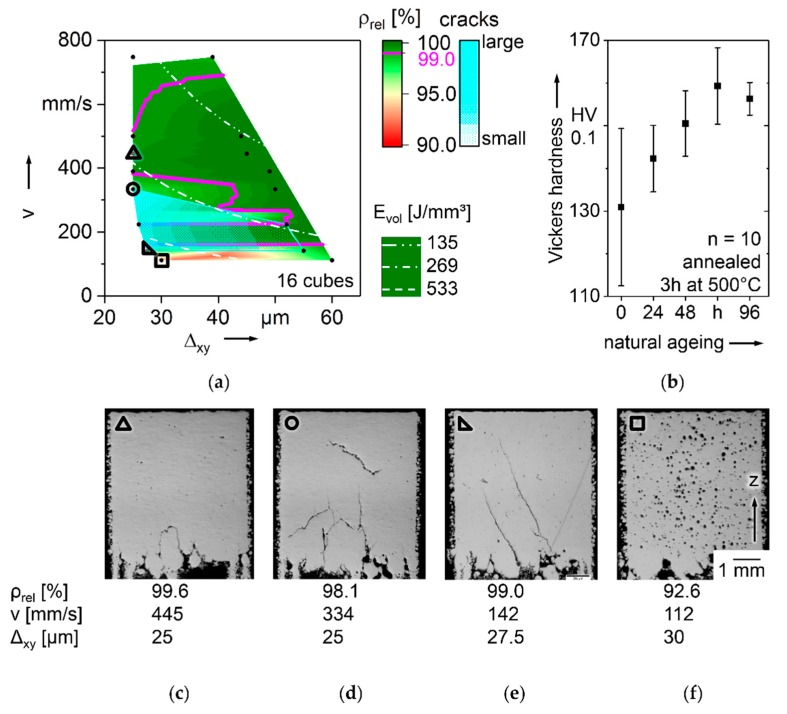
LBM process map of Al-Cu-Mg-Ti powder mixture, all particles < 20 µm; (**a**) ρ_rel_ over v and Δ_xy_, E_vol_ isolines in white, pink line ρ_rel_ = 99%, light blue marks crack tendency; (**b**) hardness development during natural ageing of a sample built with parameter as in (**c**); (**c**) sample with few, small cracks connected to the bottom surface; (**d**) sample with many cracks throughout volume; (**e**) sample with many, large cracks throughout volume; (**f**) sample with many, spherical gas pores.

Results of experimental LBM parameter variation with the quaternary Al-Cu-Mg-Ti powder mixture are illustrated in Figure 13, in the same manner as in Figure 12. All particles are sieved < 20 µm and dry coated with SiO_x_ nanoparticles. In addition to the types of defects described above for the binary mixtures, e.g., increased gas porosity in samples with very high E_vol_, exemplified in Figure 13f, some micrographs reveal cracks. Samples with cracks are marked in translucent light blue on the process map in Figure 13a. Most cracks appear to originate from the bottom surface of the cube samples, as visible in Figure 13c–e. Obviously, heat abduction from the melt pool is impeded at the bottom surface by the powder bed. It is largely thermally insulating, compared to solid Al underlying other areas. An adaptation of LBM parameters for bottom surfaces could probably provide relief but would exceed the scope of this work. Some samples, such as Figure 13c show cracks limited to areas close to the bottom. They are considered less severe. In others, such as Figure 13d, cracks appear to initiate within the sample volume. This could probably not be moderated by adapted surface processing. Figure 13e is an example with cracks that initiate from the bottom but continue so far that it appears plausible they would have opened independently from issues of the outer surface. For further experiments, the LBM parameters used for Figure 13c are chosen as the most promising. Samples with larger dimensions parallel to the build platform are built with 1 mm stripes added to the scanning strategy, as detailed in Section 2. No significant cracks in the volume are found. A horizontally oriented cylinder of 7 mm diameter and 55 mm length reveals ρ_rel_ = 99.2% confirming appropriateness of striped scanning [72]. For hardness testing, samples are built with the parameters of Figure 13c, and annealed for 3 h at 500 °C. The evolution of hardness during natural ageing is shown in Figure 13b. After 96 h, a mean of 156.3 HV0.1 is reached and standard deviation has decreased to 3.8 HV0.1, from initially 18.4 HV0.1 immediately after quenching in ice water.

### 3.7. Chemical Homogeneity Analysis of LBM Samples via EDX and BSE

**Figure 14:**
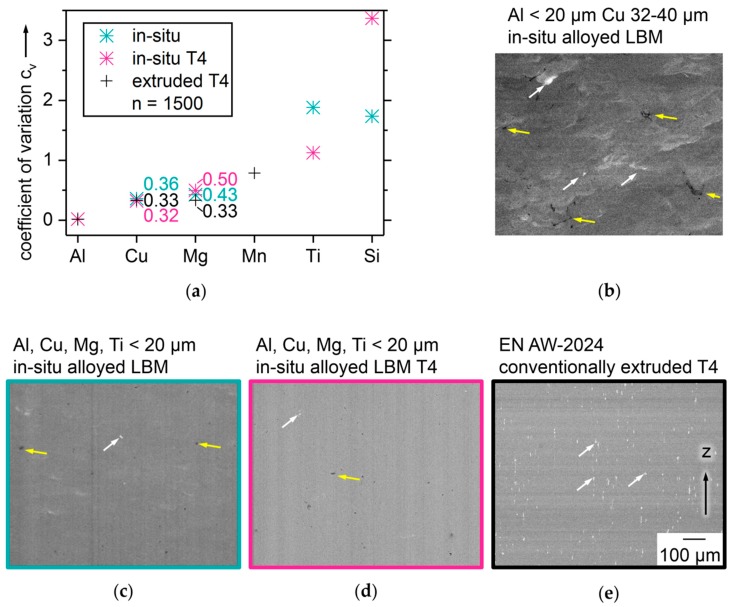
Analysis of chemical homogeneity; (**a**) coefficients of variation *C*_v_ of elements via EDX comparing in situ alloyed LBM samples made of quaternary Al-Cu-Mg-Ti mixture with particles < 20 µm and SiO_x_ as built and T4 heat-treated to a conventional EN AW-2024 T4 sample; (**b**–**e**) BSE images of samples; (**b**) LBM of binary Al-Cu, Al particles < 20 µm and Cu 32–40 µm as built; (**c**) quaternary as built; (**d**) quaternary T4; (**e**) EN AW-2024 T4 conventionally manufactured.

The results of chemical homogeneity analysis of LBM samples alloyed in situ from powder mixtures, as built, and T4 heat-treated, are illustrated in Figure 14. A sample of EN AW-2024 T4, from conventional manufacture by extrusion, is compared as a benchmark. The coefficients of variation *C*_v_ in Figure 14a show a clear tendency to increase with lower percentage of alloying elements, in all samples. The in situ alloyed LBM samples contain no Mn, while Ti and Si are not determined for the conventional sample, since they are not mandatory alloying elements in EN AW-2024 [67]. For the main alloying elements Al, Cu, and Mg, *C*_v_ are very close in the different samples. For Cu, the T4 in situ alloyed LBM sample reaches even lower C_v_ than the extruded sample. This means Cu distribution is more homogeneous than in the conventionally manufactured benchmark. For Mg and Si, *C*_v_ of the in situ alloyed samples is increased by T4 treatment, which might be caused by precipitation of intermetallic phases. The BSE images in Figure 14b–e give a more tangible impression of homogeneity. Because Cu has a higher atomic number of 29 than Al with 13, higher Cu concentrations result in brighter pixels in BSE. Mg with atomic number 12 should appear very similar to Al. Figure 14b shows irregular local increase of Cu content, in a sample in situ alloyed from a binary powder mixture with larger Cu particles, which has shown de-mixing tendencies in layer spreading. Some areas with increased Cu content are highlighted with white arrows. Over the whole cross section area, the crescent shapes of overlapping melt tracks typical for LBM can be seen. Some thin, black voids of irregular shape are highlighted with yellow arrows. The shape indicates they have probably been caused by incomplete melt coalescence. They appear more frequently in Figure 14b than in Figure 14c,d. Figure 14c shows only few spherical pores, some of which are marked with yellow arrows. Very rarely, small white spots are found. One is highlighted by a white arrow. Overall, the greyscale appears more homogeneous than in Figure 14b and the crescent shapes of melt tracks are less pronounced. Figure 14d shows a very homogeneous distribution of Cu with very few and comparatively small bright spots, crescent shaped melt tracks cannot be distinguished after T4. Still, a few spherical pores can be found as the one marked with a yellow arrow. The benchmark conventionally manufactured EN AW-2024 T4 sample in Figure 14e shows numerous white spots of increased Cu content, of which three are highlighted with white arrows. No black spots of pores are visible. The comparison of Figure 14c–e supports the interpretation of the lower *C*_v_ of Cu in Figure 14a that in situ alloyed LBM samples have chemical homogeneity very closely to or even better than the extruded sample. To investigate the changes before and after heat treatment, if and how formation of precipitation phases affects the homogeneity of elemental distribution, transmission electron microscopy might be helpful in future research.

The average elemental compositions of LBM sample and extruded sample are compared to the input from the powder mixture and the standard definition of EN AW-2024 in Table 3. The content of Mg drops from 1.57 wt% in the powder mixture to 1.02 wt% in the LBM sample. This can be explained by preferred evaporation of Mg, having a lower boiling point at 1090 °C than Al at 2470 °C. The higher boiling point of Cu at 2562 °C might have contributed to the increase of Cu content in the LBM sample and similarly for Ti boiling at 3287 °C. However, the absolute values of changes in composition should be interpreted with moderation, because the EDX measurements have been restricted to main expected alloying elements to minimize false identification [92]. Compared to the standard definition of EN AW-2024, Mn is missing in the in situ alloyed sample, because it was not added to the powder mixture. Ti content exceeds the standardized range. To reach a target composition within the standard for EN AW-2024, these deviations could be compensated much more easily than deviations of a prealloyed atomized powder by adapting the composition of the powder mixture [9,10,11]. Content of impurities in the LBM sample exceeds the limit of EN AW-2024, which could be changed by using Al powder of higher purity than the used one with 99.7 wt% as shown in Table 2. The V contained in prealloyed Ti6Al4V only amounts to 0.01% in the mixture, which is significantly below tolerated 0.05 wt% impurities per element in EN AW-2024 [67].

### 3.8. Tensile Tests

**Figure 15:**
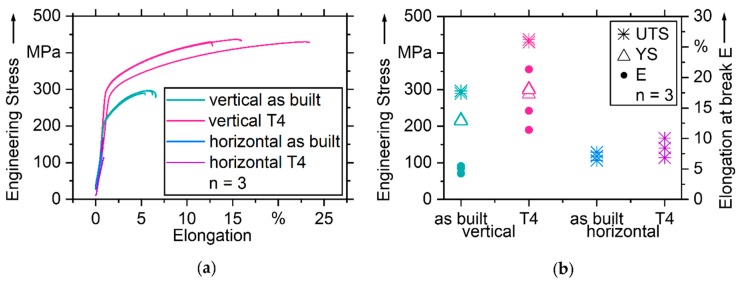
Tensile test results; (**a**) stress-strain curves; (**b**) characteristic values Ultimate Tensile Strength (UTS), Yield Strength (YS) and Elongation at break (E).

Stress-strain curves of the tensile tests are shown in Figure 15a and characteristic values ultimate tensile strength (UTS), yield strength (YS) and elongation at break (E) in Figure 15b. Twelve tensile samples are tested in four groups of three. Six samples are tested as built, six T4 heat-treated. One half has been built vertically, meaning they are pulled in parallel to buildup direction. The other half has been built horizontally and is pulled orthogonally to buildup direction. The results of the vertical specimens benefit in all characteristic values UTS, YS and E from T4. The properties of T4 treated samples are favorable at mean UTS = 432.3 MPa, YS = 296.5 MPa and E = 15.8% compared to as built UTS = 294.2 MPa, YS = 216.2 MPa and E = 4.94%. Advantages for the vertically built samples are obvious. In as built condition, vertical samples surpass the horizontal ones in UTS by a factor of 2.5, after T4 by a factor of 3.1. Horizontal samples break abruptly, so YS and E cannot be determined.

**Figure 16:**
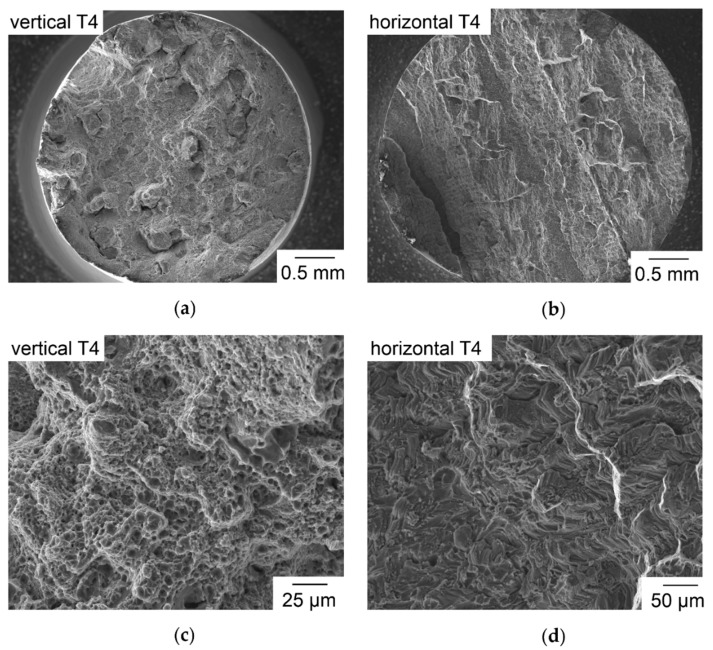
SEM images of fracture surfaces of T4 tensile specimens; (**a**) vertical built with alternating meander scan strategy; (**b**) horizontally built with alternating meander scan strategy and stripes; (**c**) detail of (**a**) in higher magnification; (**d**) detail of (**b**) in higher magnification.

Fracture surfaces of vertical T4 samples, shown in Figure 16a,c, show typical fibrous morphology of ductile plastic deformation before fracture, matching the large E of 11–21%. The smaller diameter of the sample in Figure 16a compared to Figure 16b is caused by necking during ductile deformation. In Figure 16b,d, no signs of ductile deformation are visible but sharp and edgy morphology. Figure 16b shows pronounced edges that are almost straight and continue in parallel over the whole cross section of the sample. LBM samples of prealloyed Al-Cu-Ni-Fe and Al-Cu-Mn built at P = 100 W had shown anisotropy in tensile tests, which was mainly explained by increased porosity of up to 5% in horizontal samples [11,72]. This cannot be a plausible explanation for the more pronounced anisotropy here, as a completely sectioned horizontal sample of in situ alloyed Al-Cu-Mg-Ti confirms the usefulness of the striped scanning with high ρ_rel_ = 99.2%. Fracture surfaces of horizontal Al-Cu-Mg-Ti samples reveal no increased occurrence of unmelted particles compared to the vertical samples as described in [11,72].

**Figure 17:**
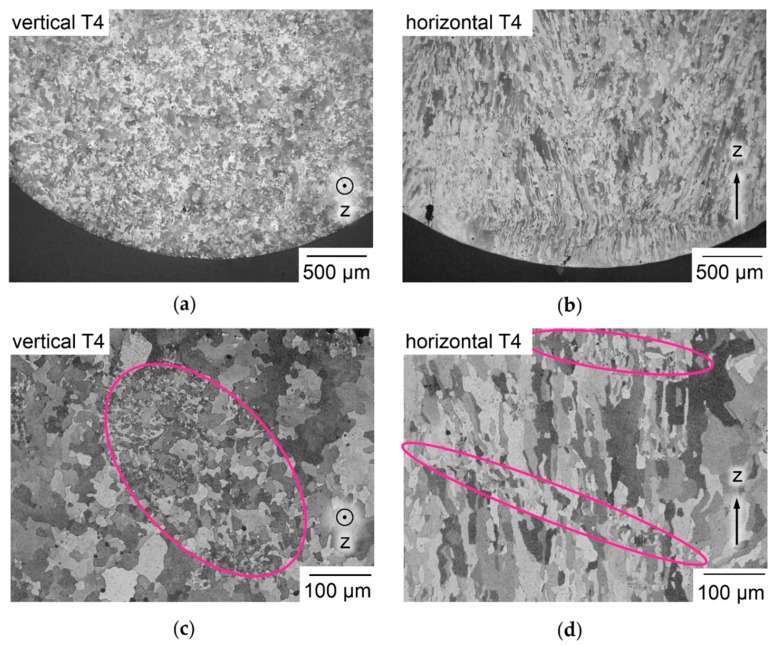
Etched micrographs of tensile specimen orthogonal to pulling direction; (**a**) vertically built specimen sectioned orthogonally to buildup direction Z with equiaxed structure; (**b**) horizontal specimen sectioned parallel to Z with elongated epitaxial grains oriented along heat flow during buildup; (**c**) larger magnification of a sample like (a); (**d**) larger magnification of a sample like (b).

The etched micrographs of horizontal and vertical samples, shown in Figure 17a–d, reveal a finer microstructure than in [11,72], which may be attributed to the Ti content known to cause grain refinement [69]. In the tested samples, Ti exceeds the limit of commercial alloy EN AW-2024, as discussed in Section 3.7 and listed in Table 3. Refined grain structure of an LBM Al-Cu-Mg alloy can lead to embrittlement, as described in [55]. Grains appear to be elongated along Z, as frequently described for additively manufactured materials, which are strongly affected by the mostly vertical heat abduction from the melt pool and epitaxial solidification with grain growth continuing over multiple Δ_z_ [93]. The grain structure of four in situ alloyed Al-Cu-Mg-Ti T4 samples is analyzed in parallel and orthogonal to buildup direction Z in the style of ISO 643 [78]. The grain size determined in Z direction as a mean of 849 grains is 30.0 µm, orthogonal to it as a mean of 3776 grains it is 16.7 µm. The mean aspect ratio is 1.8, which is low compared to the visual impression of Figure 17b,d. This may result from the method of ISO 643, in combination with the irregular grain shapes especially in planes parallel to Z, e.g., in Figure 17b,d, characterized by concave cross sections and interlocking of neighboring grains. The true dimensions of grains in 3-D must be expected larger than the determined values. Another remarkable feature is the apparently random occurrence of zones with much finer grains, e.g., the zones marked in pink ellipses in Figure 17c,d. These zones of finer grains appear to have irregular size, shape and distribution following no obviously discernible pattern in sections parallel as well as orthogonal to the buildup direction Z.

The preferred orientation of grain boundaries in parallel to Z might be a major contributing factor to the lack of ductility in the horizontal samples. The accumulation of alloying elements and impurities in grain boundaries can promote intragranular fracture that may explain the cliffy shapes in Figure 16d [94]. To change the uniformity of grain orientation, a less homogeneous temperature gradient during LBM could be realized by adapted scanning strategies.

## 4. Conclusions

It is shown that metal powder mixtures with density ratio of 3.3, as in Al-Cu, can be stabilized against segregation by using only particles < 20 µm that have been dry coated with SiO_x_ nanoparticles. Microparticle sizes > 20 µm result in pronounced de-mixing. Experimental segregation results with varied particle size fractions are contrasted to the theory that segregation in vibrated bulk powder is determined by the negative correlation of particle mobility with local concentration [37]. The most plausible explanation appears to be that effectiveness of mass density is decreased in free-flowing metal particles < 20 µm compared to larger particles. The application of stable powder mixtures for in situ alloy creation in LBM is demonstrated on the example of Al-Cu-Mg-Ti mixed from four powders with different chemical composition. Homogeneity of chemical elements in LBM samples determined via EDX and BSE measures up to, and partially surpasses, that of similar alloy EN AW-2024 manufactured conventionally by extrusion. Tensile test results underline homogeneity of the material in situ alloyed in LBM and point out the significance of grain structure in the chosen alloy system. Because the employed main effects, decreasing relevance of mass with smaller particles and lowered attraction between microparticles dry coated with nanoparticles, are based on geometry, the demonstrated approach for in situ alloy creation is expected to be transferrable to other alloying components, nanoparticles, and powder bed technologies, e.g., Electron Beam Melting and Binder Jetting [47].

## 5. Patents

A patent application resulted from the work reported in this manuscript [95].

## Figures and Tables

**Table 1:** Summary of literature on LBM in situ alloying of microscaled powder mixtures.

ρ_A_/ρ_B_	1.13	1.15	1.15	≈1	1.90	2.01	2.32	3.30
	[17]	[18]	[19,20]	[21]	[22]	[23]	[24]	[25,26]
**A-B**	Ni-Fe	Al-Si	Al-Si	HX-WC	Ti-Nb	Ti6Al4V-Cu	Ti6Al4V-Mo	Al-Cu
**A/B [wt%]**	36/63	99.4/0.6	88/12	95/5	58.2/38.6	98.6/1.4	90/10	95.5/4.5
				90/10				60/40

**Table 2:** Chemical composition of powders in wt% measured by XRF.

	Al	Cu	Mg	Ti	V	Fe	Si	Zn
Al	99.7					0.13		0.03
Cu	99.98					0.02		
AlMg50	48.4		51.3			0.13	0.23	
Ti6Al4V	5.34		0.17	90.7	3.61	0.24		

**Table 3:** Chemical composition in wt% of quaternary powder mixture calculated from mixture composition and XRF measurements of single powders, compared to in situ alloyed LBM sample measured by EDX, extruded sample measured by EDX and standard definition of EN AW-2024 [67].

	Al	Cu	Mg	Mn	Ti	V	Fe	Si	Zn	Impurities
Powder mixture	93.72	4.00	1.57		0.13	0.01	0.13	0.16	0.03	0.26
LBM sample	93.79	4.66	1.02		0.33			0.2		
Extruded sample	93.6	4.36	1.25	0.78						
EN AW-2024 [67]	bal.	3.8–4.9	1.2–1.8	0.3–0.9	<0.15		<0.5	<0.5	<0.25	<0.15

**Figure A1:**
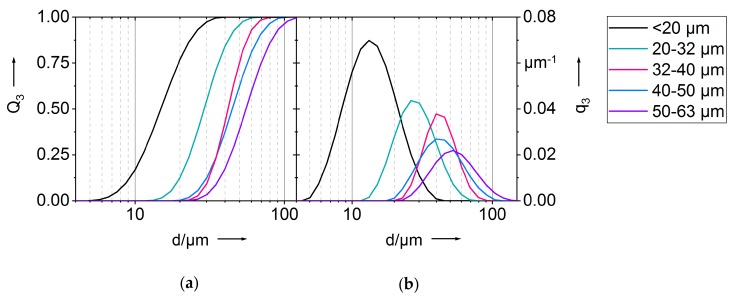
Particles size distributions (PSD) of sieved powder fractions according to ISO 9276-1 [96], 95 wt% Al, 5 wt% Cu; (**a**) cumulative particle weight fraction Q_3_; (**b**) weight specific particle size distribution density q_3_.

## Appendix A

The particle size distribution resulting from powder sieving is examined via laser diffraction particle size analysis on a Mastersizer 2000 (Malvern Instruments Ltd., Malvern, UK), with dry air dispersing. Resulting characteristic weight percentiles are listed in Table A1. Cumulative particle weight fraction, Q_3_, and weight specific particle size distribution density, q_3_, are illustrated in Figure A1. Powder fractions sieved to particle sizes of < 20 µm, 20–32 µm and 32–40 µm show distinct peaks. In comparison, the fractions sieved to 32–40 µm, 40–50 µm and 50–63 µm overlap more and differ mainly in the width of distribution. All used powders have spherical particle shape, as shown in SEM images in Figure 5, so both sieving and laser diffraction size analysis should have neglectable dependence on particle orientation.

**Table A1:** Characteristic weight percentiles of sieved powder fractions, 95 wt% Al, 5wt. % Cu, all in micrometers.

	<20	20–32	32–40	40–50	50–63
d_10,3_	8.78	19.2	30.5	28.9	35.7
d_50,3_	14.9	30.0	41.9	44.7	55.3
d_90,3_	24.4	43.5	57.5	68.4	85.0
